# Flat midsubstance of the anterior cruciate ligament with tibial “C”-shaped insertion site

**DOI:** 10.1007/s00167-014-3058-6

**Published:** 2014-05-20

**Authors:** Rainer Siebold, Peter Schuhmacher, Francis Fernandez, Robert Śmigielski, Christian Fink, Axel Brehmer, Joachim Kirsch

**Affiliations:** 1 HKF: Center for Hip–Knee–Foot Surgery, ATOS Hospital Heidelberg, Bismarckstrasse 9-15, 69115 Heidelberg, Germany; 2 Institute for Anatomy and Cell Biology, INF, Ruprecht-Karls University, Heidelberg, Germany; 3 Sportsclinic Austria, Tivoli Ost, Olympiastr. 39, 6020 Innsbruck, Austria; 4 Orthopaedic and Sports Traumatology Department, Carolina Medical Center, Pory 78, 02-757 Warsaw, Poland; 5 Institute for Anatomy Lehrstuhl I, Friedrich-Alexander-University Erlangen–Nürnberg, Krankenhausstr. 9, 91054 Erlangen, Germany

**Keywords:** ACL, Flat, Ribbon, Tibial insertion, “C”-shaped, Midsubstance

## Abstract

**Purpose:**

This anatomical cadaver study was performed to investigate the flat appearance of the midsubstance shape of the anterior cruciate ligament (ACL) and its tibial “C”-shaped insertion site.

**Methods:**

The ACL midsubstance and the tibial ACL insertion were dissected in 20 cadaveric knees (*n* = 6 fresh frozen and *n* = 14 paraffined). Magnifying spectacles were used for all dissections. Morphometric measurements were performed using callipers and on digital photographs.

**Results:**

In all specimens, the midsubstance of the ACL was flat with a mean width of 9.9 mm, thickness of 3.9 mm and cross-sectional area of 38.7 mm^2^. The “direct” “C”-shaped tibial insertion runs from along the medial tibial spine to the anterior aspect of the lateral meniscus. The mean width (length) of the “C” was 12.6 mm, its thickness 3.3 mm and area 31.4 mm^2^. The centre of the “C” was the bony insertion of the anterior root of the lateral meniscus overlayed by fat and crossed by the ACL. No posterolateral (PL) inserting ACL fibres were found. Together with the larger “indirect” part (area 79.6 mm^2^), the “direct” one formed a “duck-foot”-shaped footprint.

**Conclusion:**

The tibial ACL midsubstance and tibial “C”-shaped insertion are flat and are resembling a “ribbon”. The centre of the “C” is the bony insertion of the anterior root of the lateral meniscus. There are no central or PL inserting ACL fibres. Anatomical ACL reconstruction may therefore require a flat graft and a “C”-shaped tibial footprint reconstruction with an anteromedial bone tunnel for single bundle and an additional posteromedial bone tunnel for double bundle.

## Introduction

A detailed understanding of the anterior cruciate ligament (ACL) is the basis for anatomical ACL reconstruction. Many cadaver studies have been performed to evaluate its midsubstance size and shape and its tibial insertion in the fossa of the area intercondylaris anterior.

Most authors described the tibial ACL insertion to be oval, with the insertion of the anteromedial (AM) bundle in the AM aspect with direct relation to the medial tibial spine. The insertion of the posterolateral (PL) bundle was reported to be in the PL aspect of the ACL footprint close to the lateral tibial spine in front of the posterior root of the lateral meniscus [7, 11, 17, 28, 30, 32, 33]. Most anatomical studies were performed using paraffined specimen. The tibial attachment was reported to be an average of 10–11 mm wide and 17–18 mm long [4, 11, 14, 15] with an average area of 136 ± 33 mm^2^ [17]. Arnoczky et al. [4] found that the ACL fans out anteriorly beneath the transverse meniscus ligament and that a few fascicles of the anterior aspect of the ACL may blend with the anterior attachment of the lateral meniscus as may do some posterior fibres of the ACL with the posterior attachment of the lateral meniscus.

Based on above descriptions, the tibial ACL insertion seemed to be well described. However, recent exciting studies reported the femoral “direct” insertion of the ACL to be long and flat [20, 23, 31] and the midsubstance to be of similar flat shape [23, 24]. In concordance, R. Smigielski (“The Ribbon Concept of the Anterior Cruciate Ligament”. Presentation at the ACL Study Group Meeting 2012, Jackson Hole, Wyoming, USA) recently reconfirmed the above findings and described the ACL to be a “ribbon” and the tibial ACL insertion to be “C”-shaped. A limitation of this study was that all dissections have been performed without magnification which might allow for a systematic error during dissections. On the other hand—when reconfirmed—a flat ACL morphology and “C”-shaped insertion would have a very important impact on graft shape and bone tunnel positioning in anatomical ACL reconstruction.

The purpose of this anatomical cadaver study was to investigate the macroscopic appearance of the midsubstance shape of the ACL and its bony tibial “C”-shaped insertion site in fresh frozen and paraffined knee specimen using magnifying lenses.

## Materials and methods

Twenty human cadaveric knees (*n* = 6 fresh frozen and *n* = 14 paraffined) were used for this anatomical study. After a standard medial arthrotomy and removal of the patella, the ACL and PCL were exposed. The collateral ligaments and posterior soft tissue structures were kept for stability. An important key to the dissections was to first remove the synovial layer of the anterior horn of the lateral meniscus and to follow its shiny fibres down to its bony insertion in the central aspect of the area intercondylaris anterior. The overlaying fat pat between the insertion of the lateral meniscus and the crossing ACL was carefully removed. The midsubstance of the ACL was cleaned from surrounding synovial and fat tissue distally to its tibial insertion. In the paraffined specimen, the soft tissue structures had a yellow-like colour and were relatively stiff and hard to be discriminated by eyes. In contrast, the fresh frozen specimen was soft, and the ACL, fat pat and synovia kept their natural colours which made the dissections much easier. To ensure correct dissections, magnifying lenses were used for all steps of the dissections and all specimens (Carl Zeiss Jena, Germany).

**Fig. 1:**
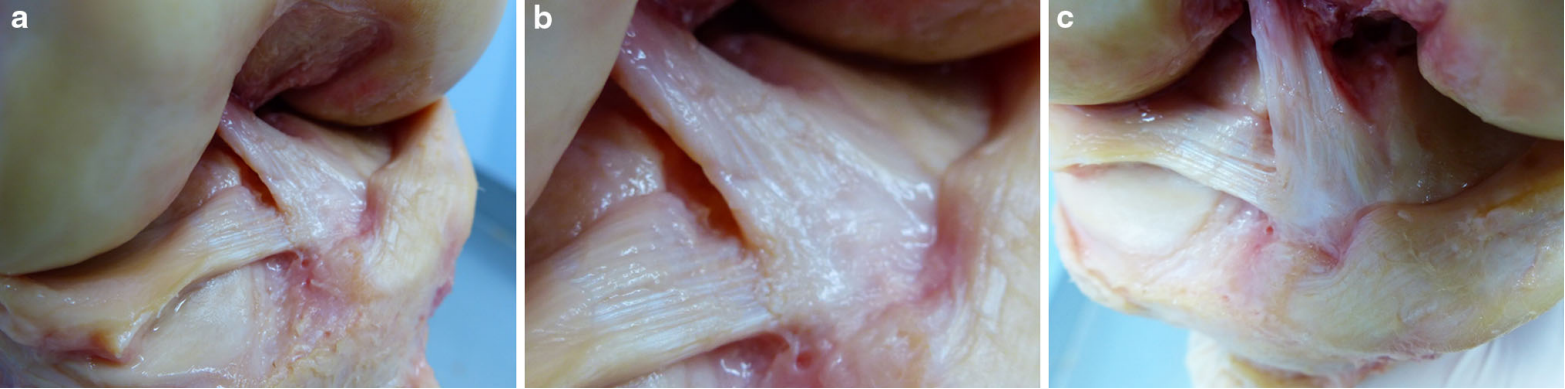
**a**–**c** Anterior horn of the lateral meniscus diving underneath the ACL; medial meniscus inserting right in front of the ACL; *AH* anterior horn of the lateral meniscus, *MM* anterior horn of the medial meniscus

The medial femoral condyle was removed to have better access to the ACL. Flexion of the knee was avoided for not to twist the ACL pretending an “oval” cross-sectional area and bundles (Fig. 1a–c). Instead, the knee was brought to full extension, which straightened the ACL fibres. Then, the ACL was temporary frozen with a standard ice spray for sports injuries and was carefully cut with a sharp blade at midsubstance. By freezing the ACL, it kept its shape. The frozen midsubstance was then “sliced” step by step and perpendicular to the longitudinal axis towards its tibial insertion. All dissections were performed by the first author, and all observations were reconfirmed by the co-authors, who watched and assisted the dissections. Morphometric measurements were performed directly at the specimen using callipers and on digital photographs.

Knees with severe osteoarthritic changes (Grades III and IV according to the Outerbridge classification [29] or damage to the ACL were excluded. Demographic data of the donors are presented in Table 1. The study was performed according to the ethical standards of the World Medical Association Declaration of Helsinki, Ethical Principles for Medical Research Involving Human Subjects.

**Table 1:** Demographic data of the donors displayed as median and range (min–max)

Gender	Side	Age (years)	Height (cm)	BMI (units)	Weight (kg)
10 female	13 right	78	166	22.8	63
7 male	7 left	(62–108)	(155–175)	(16.3–28.2)	(50–75)
3 N.A.					

### Statistical analysis

Median and range (min–max) were calculated for all donor characteristics. As the information on the smallest and largest anatomical measurements is very important for individual planning of anatomical ACL reconstructions, morphometric measurements were analysed descriptively. Therefore, all data of the morphometric measurements were tested for deviation from the normal distribution using Kolmogorov–Smirnov tests and Box-and-Whisker plots. For the Kolmogorov–Smirnov tests, a *p* value ≤0.05 was considered significant. Due to the normal distribution of all morphometric variables (*p* > 0.05) in this study, median, standard deviation (SD) and range (min–max) were calculated for all outcomes. As no group comparisons were conducted, a sample size calculation was not necessary. All analyses were conducted with SPSS 21 Statistics (IBM Corporation, Armonk, USA).

## Results

**Fig. 2:**
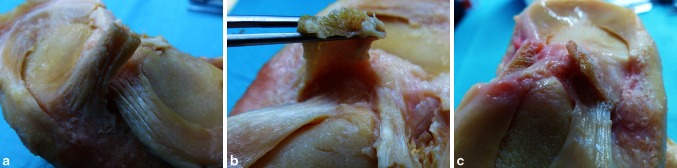
**a**–**b** ACL removed from all surrounding soft tissue and cut-off at midsubstance. In this specimen, the anterior horn of the lateral meniscus did not blend into the ACL but inserted completely posterior to the anterior “C”-shaped part of the ACL insertion. **c** Anterior fibres of the lateral meniscus blend in the anterior “C”-shaped part of the ACL insertion (most common)

The midsubstance of the ACL was flat and thin regardless of the conservation method of the specimen (paraffined or fresh frozen). When cut perpendicular to the long axis at midsubstance, it resembles a “ribbon”-like ligament with a mean width of 9.9 mm, thickness of 3.9 mm and cross-sectional area of 38.7 mm^2^ (Fig. 2a–c). Five millimetre close to the tibial ACL insertion, the mean width was 11.9 mm and thickness 3.5 mm.

**Fig. 3:**
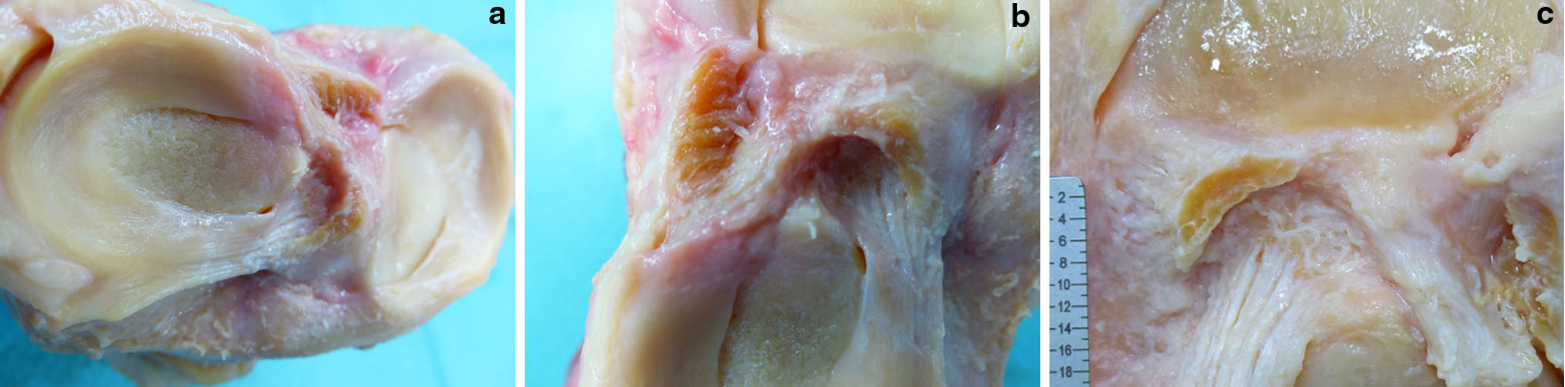
**a**–**c** ACL cut just above the tibial insertion with the “C”-shaped ACL insertion and the lateral meniscus forming a “rain-drop”-like shape. The ACL formed a “ring” structure with the lateral meniscus

The tibial ACL insertion is “C”-shaped from along the medial tibial spine to the anterior aspect of the anterior root of the lateral meniscus around a central and PL area (Fig. 3a–c). It has a mean width (length of the “C”) of 12.6 mm and thickness of 3.3 mm. The most anterior part of the “C” is a mean of 9.3 mm in the mediolateral direction, and the medial part of the “C” along the medial tibial spine is a mean of 11.4 mm in the anteroposterior direction. This corresponded to the mean anteroposterior (ap) width of 11.8 mm of the anterior horn of the lateral meniscus. The most posteromedial fibres of the “C”-shaped insertion site are a mean of 2.7 mm anterior to the tuberculum intercondylare mediale.

There are no central inserting ACL fibres, and there is no PL tibial ACL insertion. The posterior ACL fibres of the “C” are inserting medially along the medial tibial spine and were therefore named “posteromedial (PM) fibres” by the authors (Fig. 2a–c).

Details of the morphometric measurements, in particular the range with the minimal and the maximal values is displayed in Table 2.

**Table 2:** Morphometric measurements (mm) and cross-sectional area (mm^2^) of midsubstance and tibia ACL insertion displayed as mean ± standard deviation (SD), range (min–max) and median

Measurements of ACL	mean ± SD (range)	median
Width at midsubstance	9.9 ± 1.5 (7.0–12.7)	10.3
Thickness at midsubstance	3.9 ± 0.7 (2.8–4.9)	3.9
Cross-sectional area of midsubstance	38.7 ± 7.7 (20.3–51.5)	39.1
Width 5 mm proximal to tibial insertion	11.9 ± 1.1 (10.3–14.0)	11.7
Thickness 5 mm proximal to tibial insertion	3.5 ± 0.9 (2.3–5.9)	3.2
Width (length) of tibial “C”-shaped insertion	12.6 ± 2.3 (7.7–16.3)	12.7
Thickness of tibial “C”-shaped insertion	3.3 ± 0.4 (2.5–3.9)	3.3
Area of complete tibial insertion	110.9 ± 14.7 (80.1–133.1)	112.4
Area of direct “C”-shaped insertion	31.4 ± 7.2 (18.5-45.0)	30.4
Area of indirect insertion	79.6 ± 12.7 (53.7-107.7)	78.5
AP length of anterior horn lateral meniscus	11.8 ± 1.8 (8.4-15.5)	11.7
AP length of “C” along medial tibial spine	11.4 ± 2.0 (7.6-15.6)	11.3
Anterior (medial–lateral) of “C” (medial–lateral)	9.3 ± 1.6 (7.9-13.5)	8.6
Distance most posteromedial ACL insertion to tuberculum intercondylare mediale	2.7 ± 0.8 (0.8-3.8)	2.9

### Lateral meniscus

The outer fibres of the anterior and posterior horn of the lateral meniscus blend with the “C”-shaped ACL insertion like a belt, and together, they form a complete “raindrop-like” ring structure (Figs. 2a–c, 3b, c). The centre of the “C” is the place of the wide bony insertion of the anterior root of the lateral meniscus (Fig. 3a–c). It is covered by fat tissue and overpassed by the flat ACL ligament from anterior (Figs. 1a–c, 2b).

### Direct and indirect tibial ACL insertion

**Fig. 4:**
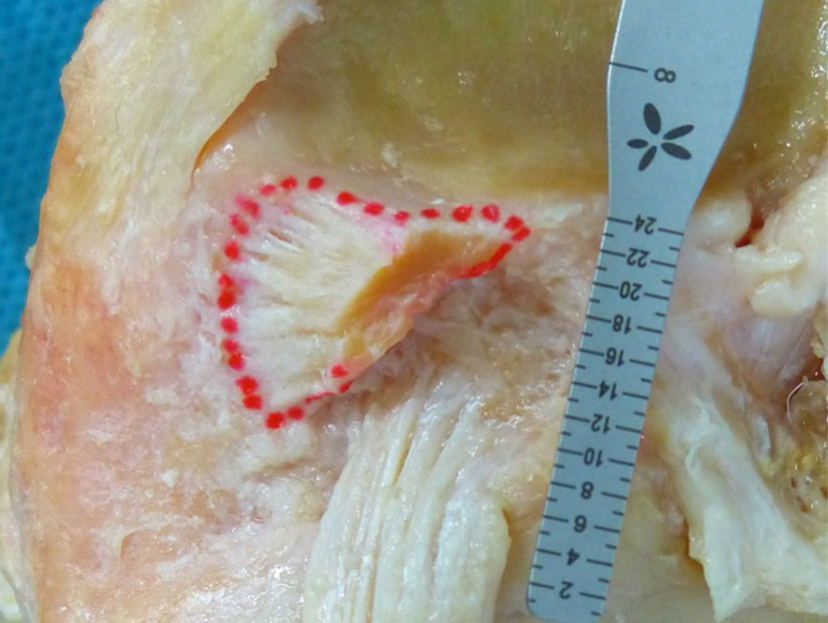
Tibial ACL footprint with it’s direct “C”-shaped and “ribbon-like” insertion site and its indirect fibres which fan out anteriorly forming a “duck-foot” (*red dots*)

Macroscopically, the tibial insertion can be divided into a “direct” and “indirect” part. The “direct” insertion is the narrow but long “C”-shaped attachment of the midsubstance fibres with an area of 31.4 mm^2^, and the “indirect” part is the anteriorly and broader attachment of the “fan-like extension” fibres with an area of 79.6 mm^2^ (Figs. 3c, 4).

The “indirect” fibres extended from the direct insertion site anteriorly and broadly spread (“fan out”) towards the anterior rim of the tibial plateau. Both insertions together form a “duck-foot-like” bony ACL footprint with a combined area of 110.9 mm^2^. An overview on morphometry of the femoral, midsubstance and tibial ACL is given in Table 3.

**Table 3:** Overview on morphometry of the femoral, midsubstance and tibial ACL from the recent literature

	Author	Width (mean)	Length (mean)	Insertion area
Femoral	Smigielski et al. (2012)	16.0 mm	3.5 mm	Direct female 52 mm^2^; male 55 mm^2^
	Mochizuki et al. [24]	15.2 mm	4.7 mm	Direct 65 mm^2^
	Iriuchishima et al. [19]			Direct 60.1 mm^2^
	Mochizuki et al. [23]			Direct 50.8 mm^2^; indirect 91.4 mm^2^; complete 142.2 mm^2^
	Sasaki et al. [31]	17.7 mm	5.0 mm	Direct 88 mm^2^
Midsubstance	Results of this study	11.9 mm	3.5 mm	37.0 mm^2^
	Smigielski et al. (2012)	11.4 mm	3.4 mm	Female 33 mm^2^; male 38 mm^2^
	Harner et al. [17]			40 mm^2^
	Hashemi et al. [18]			46.8 mm^2^
	Iriuchishima et al. [19]			46.9 mm^2^
	Anderson et al. [3]			Female 36.1 mm^2^; male 44 mm^2^
Tibial	Results of this study	12.6 mm	3.3 mm	Direct 31.4 mm^2^; indirect 79.6 mm^2^; complete 110.9 mm^2^
	Iriuchishima et al. [19]			Complete 123.5 mm^2^

Measurements document the flat appearance of the ACL including direct insertions on femur and tibia

### ACL fibre bundles

**Fig. 5:**
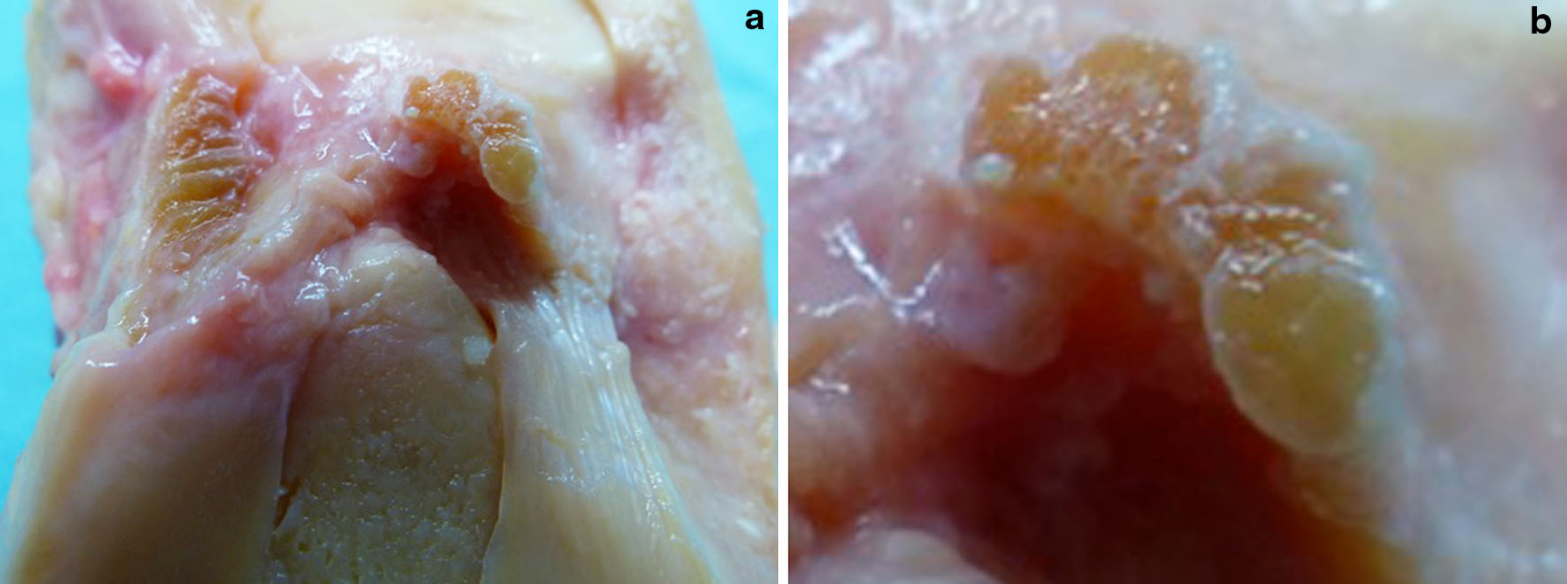
**a, b** No separate anteromedial and PL bundles could be distinguished during preparation of the ACL and its midsubstance; however, several fibre bundles were identified in some knees

**Fig. 6:**
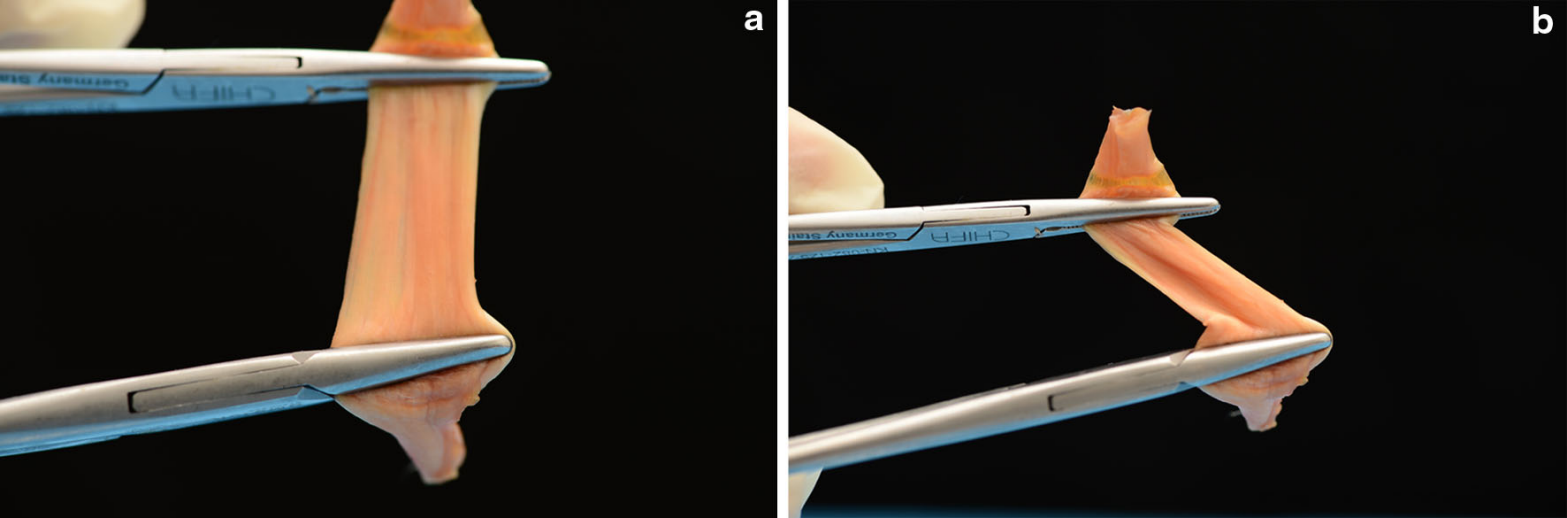
**a, b** Tendon model of “ribbon-like” ligament **a** flat, **b** twisted with bundle “effect”

The distal flat part of the ACL midsubstance consists of several small fibre bundles (Fig. 5a, b). It is impossible to clearly separate them macroscopically into an AM and PM bundle. From our observations, the appearance of macroscopic “bundles” may be created artificially by the twisted, flat ribbon-like structure of the ACL from femoral to tibial as well as the different direction of the tibial and femoral insertion site during flexion (Fig. 6a, b).

## Discussion

The most important finding of this study is that the ACL midsubstance is a flat and ribbon-shaped ligament with a “C”-shaped tibial ACL insertion. The ACL fibres insert along the medial tibial spine to the anterior aspect of the root of the lateral meniscus in the area intercondylaris anterior. Only AM and PM inserting fibres are present, no PL ones. There are also no ACL fibres in the centre of the “C”, which is the place of the bony attachment of the anterior root of the lateral meniscus.

As seen by several authors and described by R. Smigielski (ACL Study Group Meeting 2012, USA), we observed a flat and thin appearance of the ACL midsubstance [3, 17–19, 23, 24, 31]. In our specimen, the midsubstance had a mean width of 9.9 mm and thickness of only 3.9 mm which was similar to the dimensions of the “C”-shaped direct insertion site (12.6 mm versus 3.3 mm, respectively). Latter runs from along the medial tibial spine towards the anterior aspect of the anterior root of the lateral meniscus. The anteroposterior width of the lateral meniscus is very similar to the anteroposterior dimension of the tibial insertion site. The centre of the “C” is the bony insertion of the lateral meniscus, covered by fat and overpassed by the most distal fibres of the ACL from anteriorly. In contrast to previous investigators [9, 17, 22, 32, 34], we could not observe any central or PL inserting ACL fibres and no PL bundle. The posterior fibres were aligned along the medial tibial spine and were therefore named “posteromedial” (PM) fibres by the authors.

Like described for the femoral ACL insertion microscopically [20], the tibial insertion could macroscopically be divided into a “direct” and “indirect” part. The “direct” insertion was the 12.6-mm-long and 3.3-mm-thick “C”-shaped insertion of the midsubstance fibres, and the “indirect” part was the anteriorly and broader insertion of the “fan-like extension” fibres. These fibres extended from the midsubstance and broadly spread towards the anterior rim of the tibial plateau. Together, both parts formed a “duck-foot-like” bony footprint, which was described before [4]. A microscopic investigation is ongoing to support the macroscopic findings on the direct and indirect insertion.

The macroscopic separation of the ACL into bundles remains controversial. Many authors described the ACL midsubstance as a collection of individual fascicles that fan out over a broad flattened area with no histological evidence for two separate bundles [4, 8, 9, 21, 27, 35]. Our dissections reconfirm above findings, and even with magnification lenses, no separate AM and PL bundles could be distinguished. In contrast, many other authors differentiated between two [1, 5, 6, 10–14, 16, 17, 22, 25, 30, 32, 33, 36] or even three separate ACL bundles [2, 26, 28]. Amis and Dawkins [2] reported that it was “sometimes difficult to separate the ACL into three discrete bundles ‘but’ in older specimens, the separate bundles were often obvious”. From our observations and as reported before [2, 14], the appearance of macroscopic “bundles” may be artificially created by the twisted, flat ribbon-like structure of the ACL from femoral to tibial and by the different alignment of the tibial and femoral bony attachment during flexion. When dissecting cadaver knees, preparation is usually done in flexion increasing the amount of twisting of the ACL and the impression of bundles.

Above findings would support a flat ligament and footprint reconstruction. The patella tendon and quadriceps tendon have a “natural” flat shape and can be used as is in a single-bundle technique. When using hamstrings, the tendon(s) have to be “aligned” in a flat shape. For that purpose, the double-bundle technique (flat alignment of hamstring reconstruction) is theoretically superior over the single-bundle technique (round alignment of hamstring reconstruction).

Anatomical footprint reconstruction is crucial. Mochizuki et al. [23] concluded for the femur that it is very difficult to reconstruct the fan-like indirect extension fibres by a bone tunnel; however, the midsubstance fibres (=“direct” insertion) of the ACL can be reconstructed. The same applies for the tibial side; however, the “C” shape of the tibial insertion makes anatomical footprint reconstruction very difficult. In SB ACL reconstruction, an AM bone tunnel may be favoured, and in DB, an AM and PM one.

A central or PL tibial bone tunnel placement should be avoided, as both are non-anatomical, may compromise biomechanics and can damage the insertion of the anterior root of the lateral meniscus. However, the most efficient technique for ACL reconstruction has yet to be found in prospectively designed clinical long-term studies.

A limitation of this study is that all dissections were performed by the first author. However, dissections were observed by the co-authors at any time, and magnifying lenses were used for all dissection steps. Morphometric measurements were performed using callipers and on digital photography.

Remains the question why our findings were different from previous ones? An important key is the conservation method of the specimen. In paraffined specimen, all soft tissue around the ACL has a yellow-like colour and is relatively stiff. In contrast, fresh frozen specimen is softer keeping the natural colours of the ACL, fat pat and synovia, which makes the discrimination and dissections of the structures much easier. The magnifying lenses have been extremely helpful, especially when dissecting the paraffined specimen. Another very important step was to freeze the ACL before cutting which preserved the shape of the ligament.

## Conclusion

The tibial ACL midsubstance and the tibial “C”-shaped insertion are flat and are resembling a “ribbon”. The centre of the “C” is the bony attachment of the anterior root of the lateral meniscus. There are no central or PL inserting ACL fibres. Anatomical ACL reconstruction may therefore require a flat graft and a “C”-shaped tibial footprint reconstruction with an AM bone tunnel for single bundle and an additional PM bone tunnel for double bundle.
